# The Extract of* Chrysanthemum zawadskii* var. *latilobum* Ameliorates Collagen-Induced Arthritis in Mice

**DOI:** 10.1155/2016/3915013

**Published:** 2016-10-20

**Authors:** A-Ram Kim, Hyuk Soon Kim, Do Kyun Kim, Jun Ho Lee, Young Hyo Yoo, Jeom Yong Kim, Sun Kyu Park, Seung Taek Nam, Hyun Woo Kim, Young Hwan Park, Dajeong Lee, Min Beom Lee, Young Mi Kim, Wahn Soo Choi

**Affiliations:** ^1^Department of Immunology, College of Medicine, Konkuk University, Chungju 27478, Republic of Korea; ^2^Green Cross Wellbeing Co., Ltd., Seongnam 13595, Republic of Korea; ^3^College of Pharmacy, Duksung Women's University, Seoul 01369, Republic of Korea

## Abstract

*Chrysanthemum zawadskii *var.* latilobum* (CZ) has been used for beverage or tea and also as folk medicine for the remedy of diverse inflammatory diseases. Nevertheless, the therapeutic effect of CZ on arthritis remains to be unknown. In this paper we aim to investigate the CZ's antiarthritic effect and mechanism of action both in vitro and in vivo. To assess CZ's antiarthritic effect, mouse models of type II collagen-induced arthritis (CIA) were used. Mice were used to gauge clinical arthritis index and histopathological changes. Reverse transcriptase-polymerase chain reaction (RT-PCR), western blotting, electrophoretic mobility shift assay (EMSA), and other biological methods were adopted to measure CZ's effect on arthritis and to understand the veiled mechanism of action. CZ greatly suppressed CIA, histopathological score, bone erosion, and osteoclast differentiation. Mechanistically, CZ inhibited the production of various inflammatory and arthritic mediators like inflammatory cytokines, matrix metalloproteinases (MMPs), and chemokines. Of note, CZ significantly suppressed the activation of the NF-*κ*B pathway in vivo. CZ exerted an antiarthritic effect in CIA mice by curbing the production of crucial inflammatory and arthritis mediators. This study warrants further investigation of CZ for the use in human rheumatoid arthritis (RA).

## 1. Introduction

*Chrysanthemum zawadskii *var.* latilobum* (CZ) a.k.a. “Gu-jeol-cho” in Korea is a perennial plant that grows in the mountains and fields belonging to the genus* Chrysanthemum* in the family Asteraceae. According to the traditional Chinese medicine books, “Bon-cho-kang-mok,” CZ is used for the therapy of numerous illnesses like headaches, eczema, and indigestion [1].

CZ has been reported as an herbal medicine that can treat all manner of inflammatory diseases not to mention allergy and pneumonia [2]. Due to these beneficial effects, CZ extract has been used as a traditional medicine and a tea in Asian countries like Korea and China. However, the information on the antiarthritic effect of CZ extract is not yet available.

Rheumatoid arthritis (RA), one of the common autoimmune diseases, has such characteristics as the infiltration of inflammatory immune cells, synovial hyperplasia, and damage of articular tissues and bones in joints, leading to functional disability [3]. Although it is generally acknowledged that the pathological phenomena are closely associated with various inflammatory mediators such as matrix metalloproteinases (MMPs), cytokines, and chemokines [4], RA's detailed etiology and pathogenesis still stand elusive.

It is widely accepted that MMPs and proinflammatory cytokines are closely related with joint pain in RA patients [5]. MMP-1 and MMP-3 are the specific isoforms exceedingly shown in RA patients' inflamed synovium of RA, and they degrade a wide range of extracellular matrix substrates. Several inflammatory cytokines including TNF-*α*, IL-1*β*, and IL-6 are also produced in large amount in RA joints and are decisive in prompting inflammatory symptoms in humans [6]. These cytokines associated with RA pathogenesis, in combination with RANKL (receptor activator of NF-*κ*B ligand), cause bone destruction through the activation of osteoclasts known as large multinucleated bone-resorbing cells [7].

Diversified transcription factors can govern cytokine gene expression in rheumatoid synovitis [8]. Among them is nuclear factor kappa B (NF-*κ*B) that has been acknowledged to work as an essential factor in the inflammatory process by controlling the expression of diverse proinflammatory cytokines and mediators like TNF-*α*, IL-1, IL-6, and cellular adhesion molecules [9]. Thus, for the therapeutic intervention of RA, regulating NF-*κ*B activity could be advantageous [8].

In the current study, using collagen-induced arthritis (CIA) in mice, we demonstrate the antiarthritis effect and mechanism of action of CZ extract for the first time.

## 2. Materials and Methods

### 2.1. Reagents

Bovine type II collagen and Freund's complete adjuvant were attained from Chondrex (Redmond, WA, USA); recombinant human TNF-*α* from Biosource (Camarillo, CA, USA); antibodies against MMP-1 and MMP-3 from R&D Systems (Minneapolis, MN, USA); and transcription factor probes against NF-*κ*B from Panomics (Fremont, CA, USA). Cell culture media and other culture reagents used were by Gibco RBL (Gland Island, NY, USA).

### 2.2. Plant Material

The ethanol extract of* Chrysanthemum zawadskii* var.* latilobum* (CZ) was obtained for the Green Cross Wellbeing Corporation (GCWB, Seongnam-si, Korea). The GCWB from Jeongeup-si and Jeollabuk-do, Korea, gathered the plant and Professor Youngbae Suh (Natural Products Research Institute, Seoul National University, Seoul, Korea) authenticated it. Subsequently, following the institute's standard protocol, the plant extract was formulated with dried plant. In short, the active ingredient was extracted from the stems and leaves of* Chrysanthemum zawadskii* var.* latilobum* using ethanol at 50°C in a churning water bath (BS-40, JEIO-Tech, Daejeon, Korea) and concentrated using a rotary evaporator (JEIO-Tech) at 50°C. The resulting CZ extract was then stored at 4°C. It showed roughly 20% of the extraction in respect to the weight of initial material in dry condition. A voucher specimen (GC6103A-E-048) was deposited in the GCWB and in Konkuk University. For the in vitro assays, the extract was prepared by dissolving it in dimethyl sulfoxide (DMSO) and, for the in vivo animal study, it was suspended in 5% gum arabic.

### 2.3. Generation of Collagen-Induced Arthritis (CIA) in Mice

DBA/1J mice (10 five-week-old male mice per group) were bought from the Charles River Breeding Laboratories (Kanagawa, Japan) and kept in a specific pathogen-free housing facility at Konkuk University (Seoul, Korea). After granting one week of adjustment period, the study on the animals was carried out following the guideline of the institution. This was the protocol that the Institutional Animal Care and Use Committee (IACUC) at Konkuk University approved. Immunization of mice was done intradermally at the tail by injecting 100 *μ*g type II collagen emulsified with an equal volume of Freund's complete adjuvant. 23 days after this, the mice were intraperitoneally boosted with type II collagen (100 *μ*g in 0.05 M acetic acid). Starting 23 days after the initial immunization with collagen, oral administration of CZ extract (1, 10, 100 mg/kg), indomethacin (1 mg/kg), methotrexate (0.2 mg/kg), or vehicle (5% gum arabic) was conducted once a day. Nonimmunized mice were used as a normal control. Evaluation of degree of arthritis' clinical severity in all four paws of the mice was done triple-blindly based on the already published scoring system as previously reported [10]. In sum, 0 is normal; 1 is mild, apparent swelling limited to individual digits; 2 is moderate redness and swelling of the ankle; 3 is redness and swelling of the paw including digits; and 4 is maximally inflamed limb with involvement of multiple joints. Each mouse's arthritis score was calculated by adding the scores of all four paws, with the maximum score of any mouse being 16.

### 2.4. Histological Analysis

Euthanizing mice were completed on the 41st day after the first immunization with collagen. This process was carried out by fastening the right hind paws in 4% paraformaldehyde for 3 days, decalcifying them in 10% EDTA at 4°C for 30 days, drying them out in a graded ethanol series (70–100%), cleaning them twice with xylene for 3 minutes each, and then lastly burying them in paraffin. Hematoxylin and eosin (H&E) were used to stain 5 *μ*m thick serial paraffin sections. Based on the parameters presented in an earlier report [11], scores of histopathological changes in the joints were determined. Three pathologists who were uninformed of the source of the tissues separately graded each section using the 5-point scale as previously reported [10]: 0 is normal, 1 is infiltration of inflammatory cells, 2 is mild inflammation and pannus formation, 3 is moderate inflammation and pannus formation, 4 is marked infiltration of inflammatory cells, and 5 is severe infiltration of inflammatory cells and severe cartilage diffuse.

### 2.5. Microcomputed Tomography (CT)

41 days after the initial collagen injection, mice were observed and sacrificed, and their legs were severed and set in 4% formalin. The paws of the experimental mice were examined and recreated into a three-dimensional image using 18 mm voxel size of micro-CT (SkyScan 1076; SkyScan, Antwerp, Belgium). The voltage of X-ray tube with 0.5 mm thick aluminum filter was 60 kV, and the current was 170 mA. Time of exposure was 1,180 ms. X-ray projections were acquired at an interval of 0.5 *μ* with a 360 *μ* scanning angular rotation. An automated thresholding algorithm segmented the rebuilt dataset. Three-dimensional images were recreated from the projection images with NRECON software (version 1.5.1) and CT Analyzer (version 1.7), both from SkyScan.

### 2.6. Osteoclast Formation

Entire bone marrow cells were isolated from the tibia and femur of 5-week-old Balb/c mouse by rinsing out the marrow space with *α*-MEM and then removing the red blood cells. Those cells were incubated overnight in 100 mm culture dishes of *α*-MEM supplemented with 10% FBS, 1 mM pyruvate, 1% penicillin, streptomycin, and L-glutamine solution. Nonadherent cells were collected and then cultured in *α*-MEM containing 30 ng/mL M-CSF for 3 days. Bone marrow-derived macrophages (BMMs) were harvested, and 1.0 × 10^4^ BMMs were plated per well in 96-well plate with *α*-MEM containing M-CSF (30 ng/mL) and RANKL (150 ng/mL) for 4 days, with the culture medium getting changed on day 3. TRAP-positive multinucleated cells (MNCs) with more than three nuclei were considered as an osteoclast cell.

### 2.7. Tartrate Resistant Acid Phosphatase (TRAP) Staining

MNCs were prepared with 4% paraformaldehyde for 5 min. on day 4 of differentiation. Fixed cells were made permeable with 0.1% Triton X-100 for 5 min. and stained for TRAP using the Leukocyte Acid Phosphatase Kit (Sigma-Aldrich, St. Louis, MO, USA). Images of TRAP-positive cells under the microscope were captured with DP Controller (Olympus Optical, Japan). Also, to investigate CZ's effect on osteoclast formation in CIA mice, section slides were stained using a TRAP staining kit. TRAP-positive cells in ten areas of each ankle were counted.

### 2.8. Reverse Transcriptase-Polymerase Chain Reaction (RT-PCR)

18 days after boosting CIA mice with collagen, the mice's ankles were excised. Then, their tissues were ground in liquid nitrogen and kept at −70°C. Following the manufacturer's protocol, entire RNA was separated with Trizol reagent (Invitrogen, Carlsbad, CA, USA) and went through reverse transcription using the SuperScript first-strand synthesis system (Invitrogen). The primer sequences used in PCR analysis were (forward) 5′-CCAGGTGTGGGGTGCCTGAT-3′ and (reverse) 5′-CAAACCTGGGCCTGGCTGGA-3′ for mouse MMP-1; (forward) 5′-GAACATCGATGCAGCCATTT-3′ and (reverse) 5′-AGGAGAAAACGAACATTTCA-3′ for mouse MMP-3; (forward) 5′-GGCAGGTCTACTTTAGAGTCATTGC-3′ and (reverse) 5′-ACATTCGAGGCTCCAGTGAATTCGG-3′ for TNF-*α*; (forward) 5′-ATGGCAACTGTTCCTGAACTCAAC-3′ and (reverse) 5′-CAGGACAGGTATAGATTTTTCCTTT-3′ for IL-1*β*; (forward) 5′-ATGAAGTTCCTCTCTGCAAGAGACT-3′ and (reverse) 5′-CACTAGGTTTGCCGAGTAGATCTC-3′ for IL-6; (forward) 5′-CCTCTTGCTCGTGGCTGCCT-3′ and (reverse) 5′-AGTGGCTCCTGCCCTGC-3′ for MIP-1; (forward) 5′-TCCACCACCATGCAGGTCCC-3′ and (reverse) 5′-CCAGCAGGTGAGTGGGGCGTT-3′ for MCP-1; (forward) 5′-CCTCACCATCATCCTCACTGCA-3′ and (reverse) 5′-TCTTCTCTGGGTTGGCACACAC-3′ for RANTES; and (forward) 5′-TTGGCCGTATTGGGCGCCTG-3′ and (reverse) 5′-ATCGGCAGAAGGGGCGGAGA-3′ for GAPDH.

### 2.9. Electrophoretic Mobility Shift Assay (EMSA)

Isolated nuclear extracts by a kit (Affymetrix, Santa Clara, CA, USA) were reacted with a biotin-conjugated oligonucleotide for NF-*κ*B (5′-AGTTGAGGGGACTTTCCCAGGC-3′) at 15°C for 30 min., and the mixtures were then developed on a 6% nondenaturing polyacrylamide gel. The proteins were transferred to a Biodyne B membrane (Pall Corporation, Ann Arbor, MI, USA) at 300 mA for 30 min. The shift of protein band was visualized by HRP-conjugated streptavidin.

### 2.10. Isolation of Fibroblast-Like Synoviocytes (FLS)

Based on the protocol described earlier yet with slight changes, FLS were taken out from the synovial tissues gained from RA patients. In short, synovial tissues cleaned carefully with RPMI 1640 were minced and ingested for 90 min. in RPMI 1640 with 1 mg/mL of collagenase at 37°C. On a 70 *μ*m cell strainer (Becton Dickinson, Franklin Lakes, NJ, USA), the ingested tissue was sifted, and the remained cell suspension was spun at 250 ×g for 10 min. The cell pellets were cultured in *α*-minimum essential medium (*α*-MEM) composed of 10% fetal bovine serum. Patients gave an informed consent, and the Institutional Review Board at Konkuk University authorized the experimental protocol.

### 2.11. Cell Stimulation and Western Blotting

RA FLS cells (2 × 10^5^ cells/well) were rinsed 2 days after they got incubated, and the medium replaced *α*-MEM containing 1% L-glutamine and 1% antibiotics. The cells went through a pretreatment process with CZ extract for 30 min. (including an untreated control) and subsequently stimulated with 20 ng/mL TNF-*α* for 24 hours. The cell culture media were harvested and detected for MMP-1/MMP-3 secretion by western blot assay. The media were separated by sodium dodecyl sulfate-polyacrylamide gel electrophoresis and then relocated to PVDF membranes based on standard protocols. After being blocked in TBS-T buffer (10 mM Tris-HCl, pH 7.5, 150 mM NaCl, and 0.05% Tween-20) composed of 5% skimmed milk powder or bovine serum albumin, the membrane was incubated with each specific antibody. Following the manufacturer's instructions (Amersham Biosciences, Piscataway, NJ, USA), HRP-coupled secondary antibodies and enhanced chemiluminescence were used to identify the immunoreactive proteins.

### 2.12. High-Performance Liquid Chromatography (HPLC)

The CZ extract was dissolved in 1% acetic acid and 1% acetonitrile and then analyzed using an HPLC system (Shimadzu Corp., Tokyo, Japan) equipped with a PDA/ELSD detector. CZ was separated on an YMC Triart C18 (4.6 mm × 250 mm, 5 *μ*m) at a flow rate of 1.0 mL/min. To detect 3,5-dicaffeoylquinic acid (3,5-di-o-CQA), linarin, and chlorogenic acid, the mobile phase was composed of 1% acetic acid (solution A) and 1% acetonitrile (solution B) with a linear gradient elution program for a mixture of solution A and solution B from 5% solution B to 100% solution B for 50 min.

### 2.13. Statistical Analysis

The data are submitted as the mean ± SEM based on 3 or more separate experiments. One-way analysis of variance and the Dunnett test were adopted to carry out statistical analysis. The software SigmaStat (Systat Software, Inc., Point Richmond, CA, USA) was used to perform all statistical calculations (^*∗*^ *P* < 0.05 and ^*∗∗*^ *P* < 0.01).

## 3. Results

### 3.1. CZ Extract's Effect on Collagen-Induced Arthritis (CIA) of Mice

**Figure 1:**
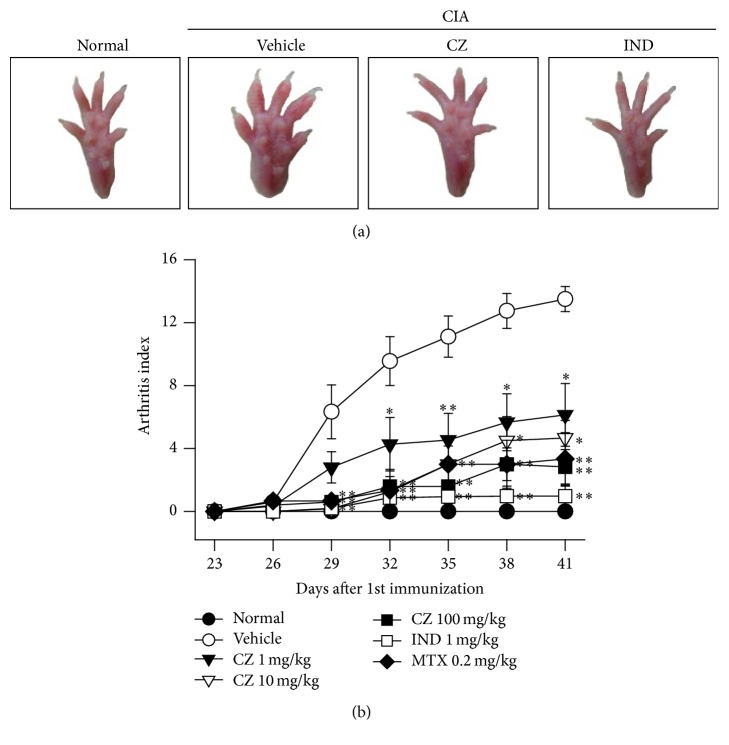
CZ extract's effect on collagen-induced arthritis (CIA) in mice. DBA/1J mice (male, 5-week-old, and *n* = 10) were immunized with 100 *μ*g of bovine type II collagen. 23 days later, mice were boosted with an intraperitoneal injection of bovine type II collagen. After the boosting injection of the collagen, CZ extract (1, 10, and 100 mg/kg), methotrexate (0.2 mg/kg), indomethacin (1 mg/kg), and vehicle were taken through the mouth once a day. The arthritis indexes of all four mouse paws were estimated according to the instructions in Section 2. Representative images of paws (a) and arthritis indexes are presented as the mean ± SEM (b) from three separate experiments. ^*∗*^ *P* < 0.05 and ^*∗∗*^ *P* < 0.01 compared with corresponding scores from vehicle-treated CIA mice. CZ, the extract of* Chrysanthemum zawadskii *var.* latilobum*; IND, indomethacin; MTX, methotrexate.

CIA is a common animal model mimicking human RA, and therefore we employed this model to test whether CZ extract inhibits CIA in mice. The arthritic symptoms were successfully induced in mice by injecting type II collagen as instructed in Section 2 (Figure 1(a)). In a preliminary experiment, CZ extract's therapeutic effect reached a plateau at a dose of 100 mg/kg CZ (data not shown). Thus, we administrated CZ extract in the dose range of 1–100 mg/kg and also used methotrexate (0.2 mg/kg) and indomethacin (1 mg/kg) for reference agents once a day for 15 days after giving a boosting shot of type II collagen on the 23rd day after giving first type II collagen shot. As shown, arthritis index was significantly decreased through the treatment of CZ extract depending on the amount of dose (Figure 1(b)). The arthritis index of the vehicle-treated control group was 13.5 ± 0.80 on day 41. The index was significantly decreased in the group treated with methotrexate, showing 3.3 ± 1.67. The decrease of indexes by CZ extract was significant and dose dependent on day 41: 6.1 ± 1.99 at 1 mg/kg, 4.7 ± 1.12 at 10 mg/kg, and 2.8 ± 1.09 at 100 mg/kg (Figure 1(b)), indicating that the potency of CZ extract at 100 mg/kg was commensurate to that of the group treated with methotrexate (Figure 1(b)).

### 3.2. CZ Extract's Effect on the Histological Changes in CIA Mice's Joint Tissues

**Figure 2:**
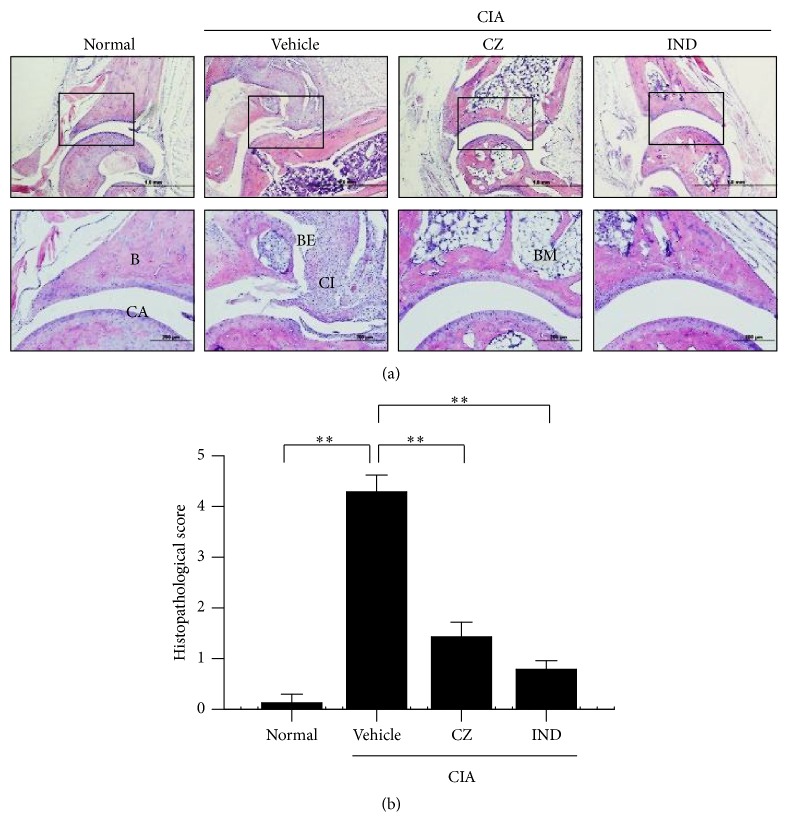
CZ extract's effect on histological changes of joints in CIA mice. (a) Histological changes of tarsal joints. Posterior paws of CIA mice were collected on day 41 as in Figure 1(b). Synovial tissues of mice's posterior paws were sectioned and stained with H&E (*n* ≥ 5). B, bone; BM, bone marrow; CA, cartilage; BE, bone erosion; CI, cell infiltration. (b) Histological scores were assessed as instructed in Section 2. Values are submitted as the mean ± SEM from three separate experiments. ^*∗∗*^ *P* < 0.01. CZ, the extract of* Chrysanthemum zawadskii *var.* latilobum*; IND, indomethacin.

We further examined CZ extract's effect on pathological changes in CIA ankle joints, such as immune cells' infiltration, cartilage damage, pannus formation, and bone decomposition. Histological sections were obtained from the hind paw joints and dyed using hematoxylin and eosin (H&E). The pathological changes such as an enormous infiltration of immune cells, cartilage damage, and bone decomposition were obvious in mice treated with vehicle compared with the normal mice (Figure 2(a)). In contrast, the pathological changes were significantly reduced in mice treated with CZ extract compared with mice treated with the vehicle (Figure 2(a)). Notably, the analysis on histological scores indicated that CZ extract (100 mg/kg) significantly suppressed the arthritic progress of hind paw joints (Figure 2(b)).

### 3.3. Effect of CZ Extract on Bone Erosion

**Figure 3:**
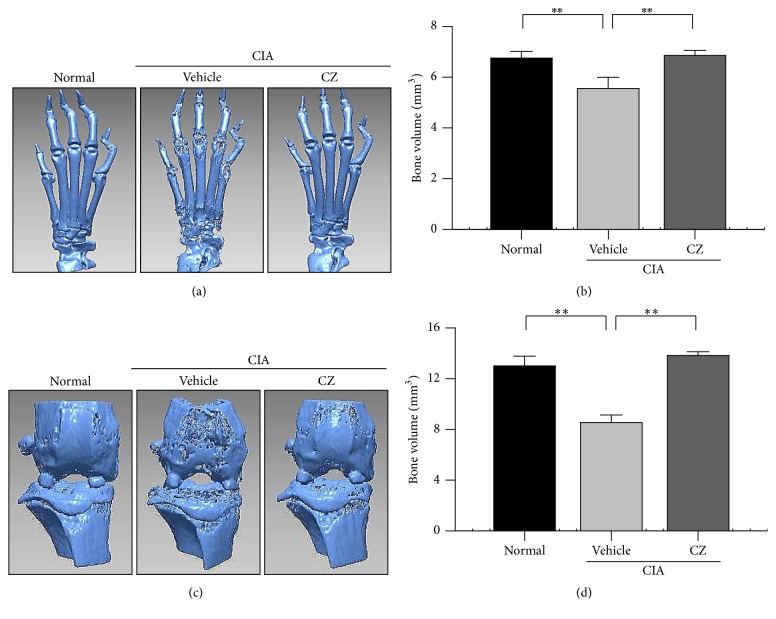
Effect of CZ extract on focal erosion of periarticular bone. The bones were analyzed by micro-CT (*n* ≥ 5). Representative three-dimensional images ((a) from hind paw; (c) from knee) and bone volumes ((b) from hind paw; (d) from knee) were obtained from three separate experiments. ^*∗∗*^ *P* < 0.01. CZ, the extract of* Chrysanthemum zawadskii *var.* latilobum*; IND, indomethacin.

Next, we tested whether CZ extract inhibits bone erosion in CIA mice using micro-CT. As Section 2 instructed, micro-CT scanned the ankle joint and paw and reconstructed them into a three-dimensional image. The bone erosion (Figure 3(a)) and decrease of bone volume (Figure 3(b)) were noticeable in the paw joints of CIA mice treated with vehicle compared with those from the normal mice. Notably, the CZ extract treatment greatly constrained those pathological bone changes in CIA mice (Figures 3(a) and 3(b)). Similar results were found in the periarticular bone of the knee joints. The erosion of the periarticular bone and decrease of bone volume were remarkable; however, CZ extract almost completely protected the mice from bone loss (Figures 3(c) and 3(d)).

### 3.4. Effect of CZ Extract on Formation of Osteoclast in CIA Mice's Joints

**Figure 4:**
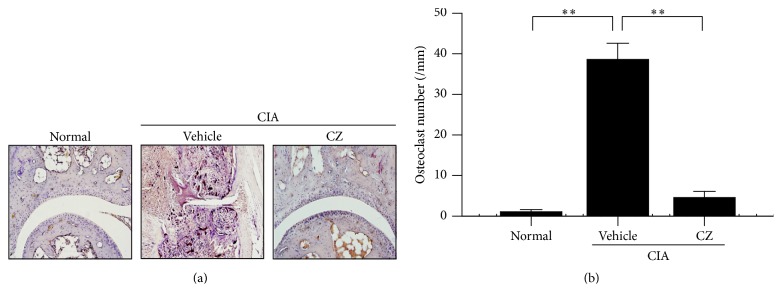
Effect of CZ extract on osteoclast formation in ankle tissues of CIA mice. On day 41, the posterior paws from CIA mice were segmented and dyed with tartrate resistant acid phosphatase (TRAP). (a) Representative images are shown. (b) The number of osteoclasts in ankle tissues from CIA mice was counted following the instruction of Section 2 (*n* ≥ 5). The number of TRAP-positive cells was presented as the mean ± SEM from three separate experiments. ^*∗∗*^ *P* < 0.01. CZ, the extract of* Chrysanthemum zawadskii *var.* latilobum*.

Multinucleated osteoclasts play a critical role in bone destruction in RA patients [12]. In this study, we investigated whether CZ extract inhibited the differentiation of osteoclasts in CIA mice's tibia. CZ extract significantly suppressed the number of osteoclasts in CIA mice's tibia (Figures 4(a) and 4(b)). These results were in agreement with the above results that the erosion of the periarticular bone and the decrease of bone volume were prevented by the administration of CZ extract.

### 3.5. Effects of CZ Extract on the Formation of MMP-1 and MMP-3 in Human RA FLS and in CIA Mice

**Figure 5:**
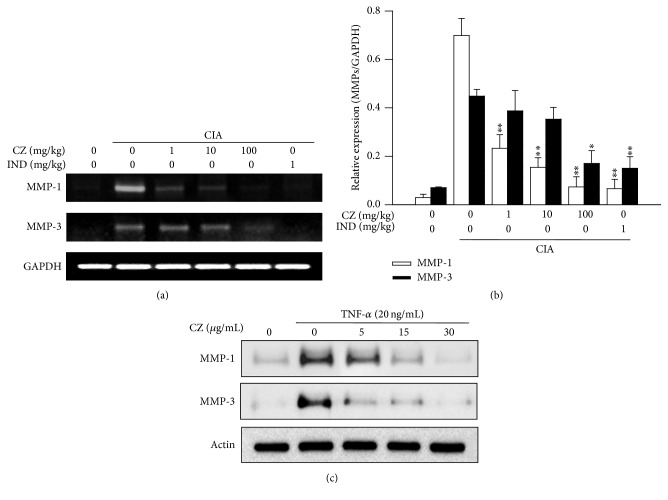
Effects of CZ extract on the production of MMP-1 and MMP-3 in CIA mice and RA fibroblast-like synoviocytes. On day 41, CIA mice's posterior paws were collected and then RT-PCR was performed. (a) Representative images are shown (*n* ≥ 5 for each group) from three independent experiments. (b) Band densities are presented as the mean ± SEM from three separate experiments. ^*∗*^ *P* < 0.05 and ^*∗∗*^ *P* < 0.01 in comparison with the scores of vehicle-treated groups. (c) The effect of CZ extract on the secretion of MMPs was measured in culture media of TNF-*α*-stimulated RA fibroblast-like synoviocytes by immunoblotting. The cells were activated with TNF-*α* (20 ng/mL) for 24 hours with and without the addition of CZ extract. Representative images are submitted from three separate experiments. CZ, the extract of* Chrysanthemum zawadskii *var.* latilobum*; IND, indomethacin.

The joint tissue destruction shown in RA patients is mainly related with the enhancement of MMP-1 and MMP-3 expression in their joints. The abundances of MMP-1 and MMP-3 in synovial fluids of RA patients are advanced compared with those of normal subjects [13]. They impair the collagenous components in cartilage and bone, which results in joint deformation and enormous pain in RA patients. Based on this notion, we next investigated the effect of CZ extract on the formation of MMP-1 and MMP-3 in CIA mice's joints. As shown in Figures 5(a) and 5(b), the expressions of MMP-1 and MMP-3 were significantly retained by CZ extract at a dose of 1 mg/kg, and the inhibition was dose dependent up to the maximum dose of 100 mg/kg of CZ. Those MMPs are mostly secreted from cytokine-stimulated FLS in synovial joint tissues [14]. We next tested whether CZ extract suppresses the secretion of MMPs from RA FLS. The secretion of MMP-1 and MMP-3 was generated from FLS with stimulation using TNF-*α* (Figure 5(c)). The secretion of MMP-1 and MMP-3 was suppressed with CZ extract depending on its dose. The secretions were almost completely blocked at a dose of 30 *μ*g/mL CZ extract (Figure 5(c)). No cytotoxicity of FLS was observed by CZ extract over the experimental dose range (data not shown).

### 3.6. Effects of CZ Extract on Inflammation-Related Gene Expression in CIA Mice

**Figure 6:**
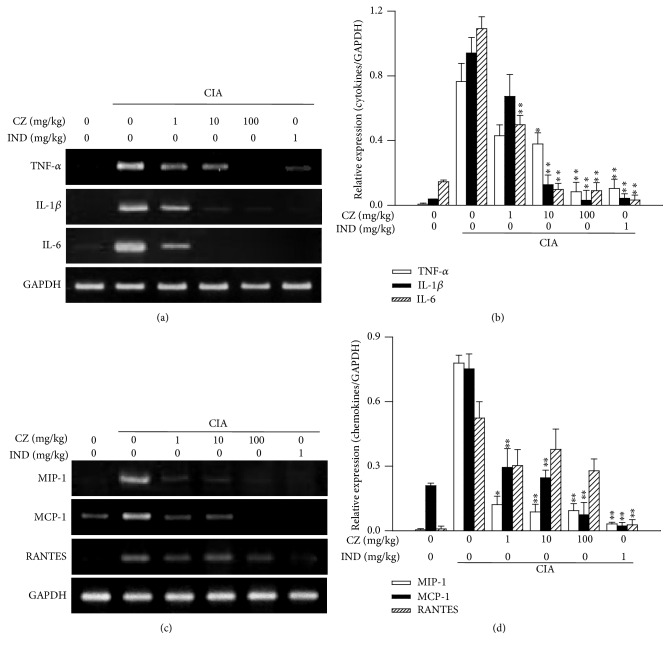
Effects of CZ extract on arthritis inflammation-related gene expression in CIA mice. The amounts of arthritis-associated mediators in CIA mice were determined by RT-PCR of CIA joint tissues. Representative images ((a) and (c)) are shown. Band densities ((b) and (d)) for the panels (a) and (c) are presented as the mean ± SEM from three separate experiments. ^*∗*^ *P* < 0.05 and ^*∗∗*^ *P* < 0.01 in comparison with the scores for the vehicle-treated group. CZ, the extract of* Chrysanthemum zawadskii *var.* latilobum*; IND, indomethacin.

Various inflammatory mediators such as cytokines and chemokines are increased in RA joint tissues and are critical to the pathogenesis of RA [5]. Thus, we investigated whether CZ extract inhibits the production of the inflammatory cytokines (TNF-*α*, IL-1*β*, and IL-6) and chemokines (MIP-1, MCP-1, and RANTES) in CIA mice. The expression of TNF-*α*, IL-1*β*, and IL-6 was remarkably increased in CIA joint tissues compared with that in normal mice (Figure 6(a)). These increases were dose dependent and significantly suppressed by the treatment with CZ extract, and, notably, the expression was almost completely blocked at 100 mg/kg CZ extract (Figures 6(a) and 6(b)). The inhibitory effect of CZ extract on the expression of MIP-1, MCP-1, and RANTES was also evident depending on dose (Figures 6(c) and 6(d)).

### 3.7. CZ Extract's Effect on the Activation of NF-*κ*B: A Transcription Factor in CIA Mice

**Figure 7:**
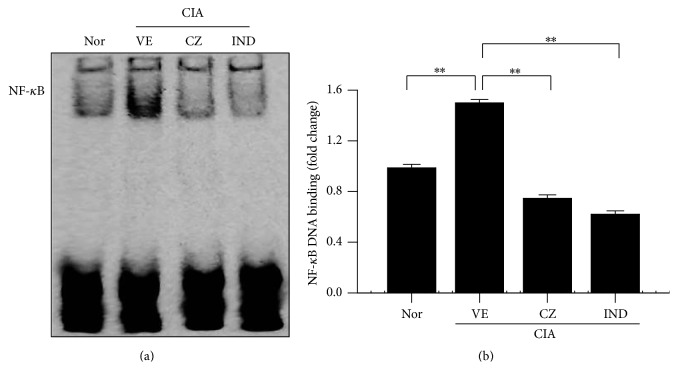
Effects of CZ extract on NF-*κ*B's activation in CIA mice. Nuclear extracts were formulated with normal or CIA ankle tissues of mice with and without CZ extract (100 mg/kg) or indomethacin (1 mg/kg). NF-*κ*B's DNA binding activity was assessed by EMSA. (a) Representative images are submitted from three separate experiments. (b) The scores of band densities are expressed as the mean ± SEM from three separate experiments. ^*∗∗*^ *P* < 0.01. Nor, normal; VE, vehicle; CZ, the extract of* Chrysanthemum zawadskii *var.* latilobum*; IND, indomethacin.

The above data suggest that CZ extract's inhibitory effect on CIA in mice may be correlated with the inhibition of numerous inflammatory factors such as MMPs, cytokines, and chemokines. It is generally believed that the activation of NF-*κ*B is crucial to the production of MMPs, cytokines, and chemokines in inflamed RA joint tissues [15]. To determine whether CZ extract suppressed the influence of NF-*κ*B in CIA mice, we measured its activity using an EMSA assay. The activity of NF-*κ*B was largely increased in vehicle-treated CIA mice (Figure 7(a)). CZ extract significantly suppressed the activity of NF-*κ*B in CIA joint tissue at a dose of 100 mg/kg, which is comparable to that of indomethacin (Figure 7(b)).

### 3.8. Analysis of Components of CZ Extract and Their Effects on RANKL-Induced Osteoclast Formation

**Figure 8:**
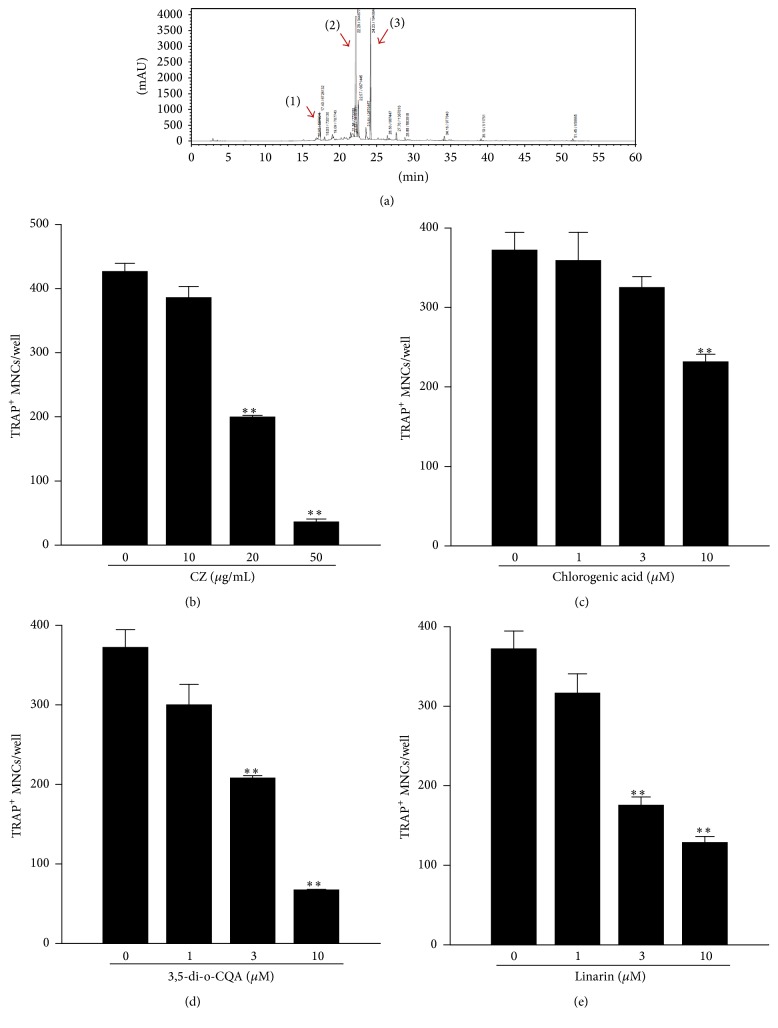
Effects of components of CZ extract on the RANKL-induced osteoclastogenesis in bone marrow-derived macrophages (BMMs). (a) HPLC profile of CZ extract and its verified components. (1) Chlorogenic acid, (2) 3,5-dicaffeoylquinic acid, and (3) linarin. ((b)–(e)) BMMs were cultured with the stated doses of CZ extract or each CZ component along with M-CSF (30 ng/mL) and RANKL (100 ng/mL) for 4 days. The number of TRAP-positive multinucleated cells with more than three nuclei was calculated. The number of TRAP-positive cells was presented as the mean ± SEM from three separate experiments. ^*∗∗*^ *P* < 0.01.

The authentic chromatographic profile for components of the CZ extract was established to reduce the probable variation of CZ ingredients in the course of each extraction procedure (Figure 8(a)). Furthermore, we identified three major components, chlorogenic acid (0.42%), 3,5-di-o-CQA (1.94%), and linarin (1.95%), in the CZ extract (Figure 8(a)). To investigate effects of those components on osteoclast formation from BMMs, we next performed RANKL-induced osteoclast differentiation with or without CZ extract or its components. Notably, the number of TRAP-positive osteoclasts was significantly decreased with CZ extract (Figure 8(b)), chlorogenic acid (Figure 8(c)), 3,5-di-o-CQA (Figure 8(d)), and linarin (Figure 8(e)) depending on the dose.

## 4. Discussion

Rheumatoid arthritis (RA) is an autoimmune disease with characteristics of chronic and systemic inflammation in synovial membranes as a result of the infiltration of inflammatory cells such as effector B cells, CD4^+^ T cells, and antigen-presenting cells, resulting in cartilage destruction and joint damage [16]. The infiltrated immune cells and synovial fibroblasts secrete various cytokines, like TNF-*α*, IL-1*β*, and IL-6, metalloproteinases (MMPs), and chemokines, such as MCP-1, MIP-1, and RANTES, in the inflamed joint tissues [10] and eventually lead to arthritis including inflammation and bone erosion.

Disease-modifying antirheumatic drugs (DMARDs) and nonsteroidal anti-inflammatory drugs (NSAIDs) are firstly suggested to treat patients with RA. NSAIDs can relieve the inflammation and pain quickly, but they do not usually prevent joint damage and have some side effects. DMARDs are used for the purpose of preventing joint damage but also have a range of side effects [17]. Recently, a category of medications known as biological response modifiers (BRMs) such as TNF-*α* inhibitors and interleukin blockers has been increasingly used to treat RA. However, there exist unwanted complications with these therapies, including fatal infections [18], and they are also very expensive. Therefore, there is a need for the development of more effective and safer medicines. Alternative herbal medicines could prove a valid approach due to their low toxicity and long-recognized activity.

The collagen-induced arthritis (CIA) mouse model is the most broadly accepted animal model for human RA. The arthritis symptoms are generated by treatment with an emulsion of complete Freund's adjuvant and type II collagen. The prominent pathogenesis is identical with critical pathological features with RA such as mononuclear cell infiltration, synovial hyperplasia, and cartilage degeneration in joints [19]. In this study, we present that, depending on the dose, the severity of arthritis symptoms is remarkably diminished by administrating CZ extract at a dose as low as 1 mg/kg (Figure 1). The effect of CZ extract at 100 mg/kg is comparable with that of treatment with methotrexate (Figure 1), a typical reference drug for RA patients. Furthermore, histopathological changes (cartilage destruction, infiltration of immune cells, and bone erosion) of the joints were significantly inhibited by the treatment of CZ extract (100 mg/kg) in CIA mice (Figure 2). Notably, the bone erosion and bone volume of foot and knee joints were also suppressed by CZ extract, as seen by micro-CT analysis (Figure 3). All in all, these findings imply that CZ extract can ameliorate the arthritis symptoms and progression in CIA mice.

Bone-erosion multinucleated osteoclasts are a key culprit cell type in the causation of bone damage in patients with RA and patients with other bone-related diseases [12]. The cells are made from the fusion of mononuclear phagocyte precursor cells in bone in order to maintain bone homeostasis. In a disease condition such as RA or osteoporosis, active osteoclasts are excessively formed by local stimulants in the lesion tissues [12]. In the present study, the number of osteoclasts was drastically reduced by the administration of CZ extract (100 mg/kg) in the ankle joints (Figure 4). These findings imply that suppressing the decrease in bone density and volume with CZ extract in CIA mice (Figure 3) is closely associated with the suppression of osteoclast formation.

Matrix metalloproteinases (MMPs) are thought to be the most critical matrix degrading enzymes in patients with RA. The MMP family comprises several members, which mediate differential impairment of individual part of the extracellular matrix. Among them, MMP-1 and MMP-3 are primary enzymes participating in joint tissue impairment. A large amount of MMP-1 and MMP-3 is observed in synovial fluid of RA patients [20]. Notably, the amount of MMP-1 and MMP-3 was significantly decreased in CIA mice's joint tissues with CZ extract treatment depending on the dose (Figures 5(a) and 5(b)). Furthermore, the increase of MMP-1 and MMP-3 in synovial fibroblasts resulted from TNF-*α* treatment and was inhibited by CZ extract (Figure 3(c)).

It is generally accepted that proinflammatory cytokines and chemokines play critical roles in worsening RA. They stimulate the production of various arthritis mediators such as MMPs and recruit immune cells to form pannus in the lesion tissues [21]. The amounts of these cytokines and chemokines in CIA joints were significantly suppressed by CZ extract in the lesion tissues (Figure 6), leading to further study into its mechanism of action for the regulation of cytokine and chemokine production. A transcription factor NF-*κ*B works as one of the main supervisors in producing inflammatory cytokine in RA [22]. The existence of activated NF-*κ*B has been detected in cultured synovial fibroblasts [23], joints of patients with RA [24], and joints of animals with CIA [25]. The activation of NF-*κ*B generates the expression of inflammatory mediators such as adhesion molecules, chemokines, and cytokines [26]. Thus, we investigated CZ extract's effect on NF-*κ*B's DNA binding activity in CIA mice's joints. In line with above results (Figure 6), NF-*κ*B's DNA binding activity was inhibited with CZ extract in CIA mice (Figure 7), suggesting that the mechanism of action of CZ extract for its antiarthritis effect is intimately related with the inhibition of the NF-*κ*B activation in arthritic joints.

Through the compositional analysis of CZ extract, we determined three major components using HPLC (Figure 8(a)). Among them, chlorogenic acid (CGA) is a natural phenolic compound which suppresses rheumatoid arthritis [27] and some inflammatory responses in macrophages [28]. Linarin is a natural flavonoid, which has been reported as a substance having antioxidant and anti-inflammatory activities [29]. Caffeoylquinic acid (CQA) derivatives are components which show antioxidant, antihistaminic, and anti-inflammatory effects [30]. In this study, we demonstrated that CGA, 3,5-di-o-CQA, and linarin also inhibited RANKL-mediated osteoclast differentiation (Figures 8(c)–8(e)). The results suggested that CGA, 3,5-di-o-CQA, and linarin of CZ extract could cooperatively ameliorate collagen-induced arthritis in mice.

## 5. Conclusion

For the first time, we manifest that CZ extract effectively suppresses synovial inflammation, osteoclast formation, and the development of CIA in mice. Mechanistically, CZ extract suppresses the expressions of inflammatory-related genes by inhibiting the activation of NF-*κ*B in joint tissues of CIA mice. Although any toxicity with the traditional use of CZ extract has not been recognized up to now, systemic experiments on its toxicity and dosage finding for the application to patients with RA are in progress for the Investigational New Drug (IND) application.
